# TASK‐1 channel blockade by AVE1231 increases vasocontractile responses and BP in 1‐ to 2‐week‐old but not adult rats

**DOI:** 10.1111/bph.15249

**Published:** 2020-09-24

**Authors:** Anastasia A. Shvetsova, Dina K. Gaynullina, Nadine Schmidt, Peter Bugert, Elena V. Lukoshkova, Olga S. Tarasova, Rudolf Schubert

**Affiliations:** ^1^ Centre for Biomedicine and Medical Technology Mannheim (CBTM) and European Center of Angioscience (ECAS), Research Division Cardiovascular Physiology, Medical Faculty Mannheim Heidelberg University Heidelberg Germany; ^2^ Faculty of Biology M. V. Lomonosov Moscow State University Moscow Russia; ^3^ Institute of Transfusion Medicine and Immunology, Medical Faculty Mannheim Heidelberg University, German Red Cross Blood Service Baden‐Württemberg—Hessen Mannheim Germany; ^4^ Institute of Experimental Cardiology National Medical Research Center of Cardiology Moscow Russia; ^5^ State Research Center of the Russian Federation—Institute for Biomedical Problems Russian Academy of Sciences Moscow Russia; ^6^ Physiology, Institute of Theoretical Medicine, Medical Faculty University of Augsburg Augsburg Germany

**Keywords:** arterial smooth muscle, K2P, membrane potential, ontogenesis, TASK‐1, vascular tone

## Abstract

**Background and Purpose:**

The vasomotor role of K2P potassium channels during early postnatal development has never been investigated. We tested the hypothesis that TASK‐1 channel (K2P family member) contribution to arterial vascular tone and BP is higher in the early postnatal period than in adulthood.

**Experimental Approach:**

We studied 10‐ to 15‐day‐old (“young”) and 2‐ to 3‐month‐old (“adult”) male rats performing digital PCR (dPCR) (using endothelium‐intact saphenous arteries), isometric myography, sharp microelectrode technique, quantitative PCR (qPCR) and Western blotting (using endothelium‐denuded saphenous arteries), and arterial pressure measurements under urethane anaesthesia.

**Key Results:**

We found mRNA of *Kcnk1*–*Kcnk7*, *Kcnk12*, and *Kcnk13* genes to be expressed in rat saphenous artery, and *Kcnk3* (TASK‐1) and *Kcnk6* (TWIK‐2) were most abundant in both age groups. The TASK‐1 channel blocker AVE1231 (1 μmol·L^−1^) prominently depolarized arterial smooth muscle and increased basal tone level and contractile responses to methoxamine of arteries from young rats but had almost no effect in adult rats. The level of TASK‐1 mRNA and protein expression was higher in arteries from young compared with adult rats. Importantly, intravenous administration of AVE1231 (4 mg·kg^−1^) had no effect on mean arterial pressure in adult rats but prominently raised it in young rats.

**Conclusion and Implications:**

We showed that TASK‐1 channels are important for negative feedback regulation of vasocontraction in young but not adult rats. The influence of TASK‐1 channels most likely contributes to low BP level at perinatal age.

**Abbreviations**
BKCa: potassium channel, calcium‐activated large conductance subfamily M α, member 1
BP: blood pressure
dPCR: digital PCR
FAM: carboxyfluorescein
HR: heart rate
K2P: two‐pore‐domain potassium channel
Kir: inwardly rectifying potassium channel
Kv: voltage‐gated potassium channel
MAP: mean arterial pressure
pD_2_: the negative logarithm of EC50
qPCR: quantitative PCR
RIN: RNA integrity number
TASK‐1: potassium channel, two‐pore‐domain subfamily K, member 3
TBSt: tris‐buffered saline with 0.1% Tween 20
TWIK‐2: potassium channel, two‐pore‐domain subfamily K, member 6

**What is already known**

- TASK‐1 channels are expressed in arteries of the pulmonary and systemic circulation.
- TASK‐1 channel blockade by AVE1231 increases contractile responses in pulmonary arteries.

**What this study adds**

- TASK‐1 channel expression is higher in arteries of 1‐ to 2‐week‐old compared with adult rats.
- TASK‐1 channels contribute to arterial vascular tone and the setting of the BP level in the early postnatal period.

**What is the clinical significance**

- The pathogenesis of cardiovascular diseases in neonates can be associated with TASK‐1 channel dysfunction.
- Potential vasopressor actions of TASK‐1 channel blockers should be considered in the recently suggested TASK‐1 channel‐targeted treatment of cardiac arrhythmia.

## 1 INTRODUCTION

Adaptive changes in the blood supply of organs are a hallmark of their growth and maturation (Štulcová, 1977). The vascular system of the immature organism is characterized by a number of structural and functional features, including, but not limited to, low arterial pressure and greatly altered mechanisms of arterial tone regulation (D. K. Gaynullina, Schubert, & Tarasova, 2019; D. Gaynullina et al., 2013; Longo & Goyal, 2013; Mochalov et al., 2018; Sofronova et al., 2016). Interestingly, a few studies addressed the role of potassium channels in tone regulation of the systemic vasculature during the early postnatal period. Thus, in the immature period, BKCa channel blockade was ineffective regarding tone regulation in rat basilar arteries (Gollasch et al., 1998), and BKCa and Kv channel blockade produced only very small effects in sheep middle cerebral arteries (Teng, Nauli, Brayden, & Pearce, 2002), in contrast to later developmental stages. In rat aorta, the effects of BKCa channel blockade also increased with maturation, but the anticontractile influence of Kv channels was higher in the neonatal period (Gomez, Ghisdal, & Morel, 2000). Recently, we showed that in rat saphenous arteries in the early postnatal period, the negative feedback regulation of vasocontraction by BKCa channels was small, but the influence of Kv1, Kir, and Kv7 channels was quite pronounced compared with adult animals (Shvetsova, Gaynullina, Tarasova, & Schubert, 2019). Thus, these data suggest that the contribution of potassium channels to arterial tone regulation in the early postnatal period varies depending on the vascular bed and/or channel type and, therefore, cannot be predicted for channels not studied yet.

There is one potassium channel family, the K2P channels, whose role in arterial tone regulation has been studied with comparatively low intensity so far. Research over the last years has changed the image of these channels from being simply leak channels to regulatory channels having important roles in the control of cell excitability (Renigunta, Schlichthörl, & Daut, 2015). Several members of the K2P channel family have been shown to be expressed differentially in various arteries (Gurney & Manoury, 2009). Most published studies have been focused on TASK‐1 channels expressed in the majority of the arteries investigated so far (Gurney & Manoury, 2009). This channel was suggested to mediate changes in arterial tone induced by pH alterations (Gardener et al., 2004) or hypoxia (Nagaraj et al., 2013; Olschewski et al., 2006) in rabbit, rat, and human arteries. Moreover, TASK‐1 channels have been proposed to be involved in human pulmonary arterial hypertension (Antigny et al., 2016; Ma et al., 2013). However, the contribution of TASK‐1 channels to the regulation of vascular tone during early postnatal development has never been addressed.

We tested the hypothesis that TASK‐1 channel contribution to arterial vascular tone and BP is higher in the early postnatal period than in adulthood. To evaluate the functional impact of TASK‐1 channels, we used AVE1231, which was initially described as a Kv1.5 channel blocker, but then demonstrated higher affinity for TASK‐1 channels (Kiper et al., 2015). To provide evidence for the specificity of the effect of AVE1231 on TASK‐1 channels in relation to its possible effects on Kv1.5 channels, we used DPO‐1, a specific Kv1 channel blocker (Lagrutta, Wang, Fermini, & Salata, 2006; Tsvetkov et al., 2016).

## 2 METHODS

### 2.1 Animals

Male Wistar rats aged 10 to 15 days (“young” in the following text) and 2 to 3 months (“adult” in the following text) were used in this study (obtained from Janvier, France, or the Institute of General Pathology and Pathophysiology, Russia). Rats were killed under CO_2_ anaesthesia by decapitation. Animal studies are reported in compliance with the ARRIVE guidelines (Percie du Sert et al., 2020) and with the recommendations made by the *British Journal of Pharmacology* (Lilley et al., 2020). Approval for the use of laboratory animals and all procedures used in this study was granted by German and Russian institutional committees on animal welfare (I‐17/17 and 93‐g, respectively). Rats have been used for research on potassium channel function in early postnatal ontogenesis in many studies (Belevych, Beck, Tammaro, Poston, & Smirnov, 2002; Gollasch et al., 1998; Shvetsova et al., 2019). Since the male body is not subjected to cyclical hormonal changes, we decided to employ a commonly used approach, namely, to explore a novel physiological mechanism only in males. The animals were provided with food and water ad libitum and housed in a room with a controlled temperature and a 12‐h light–dark cycle.

### 2.2 Wire myograph experiments

The saphenous artery, a muscle‐type artery of the cutaneous circulation, was chosen as the main object for the present study. The arteries were carefully cleaned from surrounding tissue, cut into 2‐mm‐long segments, and mounted in a wire myograph (410A or 620M, DMT A/S, Denmark) to measure isometric force. Right after mounting, the endothelium was gently denuded using a rat whisker. These procedures were performed in a solution containing (mmol·L^−1^): NaCl 145; KCl 4.5; CaCl_2_ 0.1; MgSO_4_ 1.0; NaH_2_PO_4_ 1.2; EDTA 0.025; and HEPES 5.0 (pH = 7.4).

During the subsequent experiments, arterial segments were kept in a solution containing (mmol·L^−1^): NaCl 120; NaHСO_3_ 26; KCl 4.5; CaCl_2_ 1.6; MgSO_4_ 1.0; NaH_2_PO_4_ 1.2; d‐glucose 5.5; EDTA 0.025; and HEPES 5.0. The myograph chambers were heated to 37°C and continuously bubbled with 5% CO_2_ in O_2_ to maintain pH at 7.4. Data were recorded at 10 Hz using an analogue‐to‐digital converter (E14‐140М, L‐CARD, Russia) and the PowerGraph 3.3 software (DISoft, Russia) or the PowerLab 4/30 system (ADInstruments, USA) and the LabChart software (ADInstruments, USA). Each arterial segment was stretched to 0.9d_100_ (90% of the inner diameter it would have at a transmural pressure of 100 mmHg), corresponding to maximum active force development (Mulvany & Halpern, 1977; Shvetsova et al., 2019).

At the beginning of each experiment, the following substances activating contractile and relaxing pathways were applied successively: (i) noradrenaline (10 μmol·L^−1^); (ii) methoxamine (α_1_‐adrenoceptor agonist, 1 μmol·L^−1^) and, on top after 5 min, ACh (10 μmol·L^−1^)—to confirm successful endothelium removal by the absence of a relaxation response; and (iii) methoxamine (10 μmol·L^−1^).

The experimental protocol included two sequential concentration–response relationships to methoxamine (concentration range from 0.01 to 100 μmol·L^−1^). The first relationship was started 20 min after the end of the activation procedure. Thereafter, one arterial segment was incubated for 20 min with a potassium channel blocker (AVE1231, DPO‐1, or their combination) and the other one—with an equivalent volume of the solvent (time control). Then the second concentration–response relationship was obtained (for details, see Figure 2).

To calculate active force values at each methoxamine concentration, the force value at the fully relaxed state was subtracted from all recorded values. All active force values obtained during the second concentration–response relationship (which are shown in Figure 3a–f) were expressed as the percentage of the maximum active force developed during the respective first concentration–response relationship. Presentation of data in percentage allows to eliminate variability due to differences in the size of different vessels. To estimate the initial sensitivity of arteries to methoxamine, individual concentration–response relationships were fitted to a sigmoidal dose–response (variable slope) equation using GraphPad Prism 7.0 (La Jolla, CA, USA, RRID:SCR_002798) to obtain pD_2_ (the negative logarithm of EC_50_).

### 2.3 Membrane potential measurements

Smooth muscle membrane potential was measured simultaneously with isometric force using the sharp microelectrode technique as described in our previous study (Shvetsova et al., 2019). Briefly, saphenous arteries were isolated, denuded of the endothelium, and mounted in a wire myograph (Model 301, DMT A/S, Denmark). At the beginning of each experiment, vessels were normalized (0.9d_100_ values were determined), then the activation procedure was performed, and a single concentration–response relationship to methoxamine was obtained (for details, see Section 2.2). To exclude distortion of the electrode by gas bubbles during membrane potential measurements, the experimental solution was heated to 37°C and bubbled with 5% CO_2_ in O_2_ in a separate reservoir and then supplied to the myograph chamber by a peristaltic pump (Julabo, Germany) at a rate of 2 ml·min^−1^.

Microelectrodes were made of aluminosilicate glass, filled with saturated KCl, and had resistances of 30 to 70 MΩ. The resistance of the electrode was continuously monitored by applying subthreshold electrical pulses (20‐mV amplitude and 25‐ms duration) at a frequency of 1 Hz. Membrane potential recordings were accepted if they were characterized by (i) a sharp drop of the potential on cell penetration; (ii) a stable level of the membrane potential recording for at least 30 s; (iii) a return to the zero‐potential level after electrode removal; and (iv) a similar electrode resistance before and after the measurement.

Membrane potential and force were determined under five different experimental conditions (Figure 4): (1) in the absence of any substance (Baseline 1); (2) during a methoxamine‐induced contraction adjusted to ~20% of maximum force (the appropriate concentration of methoxamine was identified based on the concentration–response relationship); (3) after washout of methoxamine (Baseline 2); (4) in the presence of 1 μmol·L^−1^ of AVE1231 or DMSO (equivalent volume); and (5) during methoxamine‐induced contraction (the same methoxamine concentration as at Step 2) in the presence of AVE1231 or DMSO.

Active force values were calculated as the percentage of the maximum force obtained during the concentration–response relationship to methoxamine.

### 2.4 Measurement of mRNA expression levels in arterial tissue by digital PCR

Endothelium‐intact saphenous arteries were quickly isolated from young and adult rats. The preparation procedure was carried out in ice‐cooled solution (for composition, see Section 2.2.). After isolation, arteries were frozen in liquid nitrogen and stored at −80°C until further manipulations. Each sample included four arteries from two young rats or one artery from an adult rat.

Arteries were homogenized using the TissueLyser (Qiagen). Total RNA was isolated with the miRNeasy Mini Kit (Qiagen) according to the manufacturer's instructions including a DNase‐treatment step. RNA concentration was measured using a Tecan Plate Reader. RNA quality was determined with a Bioanalyzer (ZMF). Only samples with high quality (RNA integrity number [RIN] >7.5) were used for further measurements.

For cDNA synthesis, 1‐μg total RNA was used in a standard protocol (SensiFAST™ cDNA Synthesis Kit; BioCat GmbH (RRID:SCR_001440), Heidelberg, Germany). First, expression of the *Kcnk1*–*Kcnk7*, *Kcnk9*, *Kcnk10*, *Kcnk12*, *Kcnk13*, *Kcnk15*, *Kcnk16*, and *Kcnk18* genes was tested in two cDNA samples from each age group of rats by quantitative PCR (qPCR) using commercial assays (TaqMan™ Gene Expression Assay, Thermo Fisher Scientific GmbH, Dreieich, Germany). Second, chip‐based digital PCR (dPCR; QuantStudio® 3D, Thermo Fisher Scientific) was performed for absolute quantification of mRNA copies per μl for the *Kcnk* genes detectable by qPCR. The TaqMan assays for *Kcnk1*–*Kcnk7*, *Kcnk12*, and *Kcnk13* were applied to all cDNA samples. After cycling, the dPCR chips were scanned for the carboxyfluorescein (FAM) signals, and the data were uploaded to the QuantStudio 3D AnalysisSuite cloud software (https://apps.thermofisher.com/quantstudio3d). Based on the fluorescence signals and statistical correction using Poisson distribution, the software enabled calculation of target copies per μl.

### 2.5 Measurement of mRNA expression levels in arterial tissue by qPCR

Saphenous arteries were isolated, carefully cleaned from surrounding tissue, cut into 8‐mm‐long segments, and quickly mounted in an ice‐cooled analogue of a wire myograph system, to remove the endothelium using a rat whisker. Endothelium‐denuded arterial segments were kept in RNA‐later solution (Qiagen) at −20°C pending further procedures. Each sample included four arterial segments, each from two young rats, or two arterial segments from one adult rat. RNA was extracted using the ExtractRNA kit (Evrogen, Russia) according to the manufacturer's instructions, processed with DNase I (Fermentas, 1,000 U·ml^−1^). RNA concentration was measured by a NanoDrop 1000 (Thermo Scientific, USA), and then all samples were adjusted to a concentration of 70 ng·μl^−1^. Reverse transcription was performed using the MMLV RT kit (Evrogen, Russia) according to the manufacturer's manual. qPCR was run in the RotorGene6000 using qPCRmix‐HS SYBR (Evrogen, Russia).

Primers used in this study were synthesized by Evrogen and had the following sequences: *Kcnk3* (forward: TGTCCATGGCCAACATGGT; reverse: GGAAGAAAGTCCAGCGCTCAT), *Gapdh* (forward: CACCAGCATCACCCCATTT; reverse: CCATCAAGGACCCCTTCATT), *Rn18s* (forward: CACGGGTGACGGGGAATCAG; reverse: CGGGTCGGGAGTGGGTAATTTG), and *Nos3* (forward: GGATTCTGGCAAGACCGATTAC; reverse: GGTGAGGACTTGTCCAAACACT). Primer efficiency was identified using the LinRegPCR software (Ruijter et al., 2009) and was in the range of 1.8–2.0. mRNA expression levels were calculated as *E* ^*−Ct*^, where *E* is the primer efficiency and *Ct* is the cycle number on which product fluorescence rose above the threshold level. These values were normalized to the geometric mean of the expression values for two housekeeping genes (*Gapdh* and *Rn18s*), detected in the same sample. To compare TASK‐1 expression between two experimental groups, all values were expressed as the percentage of the median value in the adult group. Successful endothelium removal was confirmed by about 10‐fold drop of *Nos3* mRNA content in endothelium‐denuded versus endothelium‐intact samples (Figure S1).

### 2.6 Measurement of protein abundance in arterial tissue by Western blotting

Four saphenous arteries from two young rats or two saphenous arteries from one adult rat were used for one sample. The endothelium was rapidly removed after isolation of the artery in the same way as described for qPCR. Then arterial samples were quickly frozen in liquid nitrogen and kept at −80°C till further analysis. Samples were homogenized in ice‐cold SDS buffer (0.0625 mol·L^−1^ of Tris–HCl (pH 6.8), 2.5% SDS, 10% water‐free glycerine, 2.47% DTT, and 0.002% bromophenol blue) supplemented with protease inhibitors (aprotinin 50 mg·ml^−1^, leupeptin 100 mg·ml^−1^, and pepstatin 30 mg·ml^−1^), centrifuged at 16,900 *g* for 5 min; the supernatant was kept at −20°С till further analysis. Proteins were separated by SDS‐PAGE and transferred to nitrocellulose membranes (Santa Cruz, USA) using the Trans‐Blot Turbo transfer system (Bio‐Rad). The transfer was visualized with Ponceau S staining, which was further used as loading control (Romero‐Calvo et al., 2010). Thereafter the piece of membrane (at the level of the TASK‐1 channel—approx. 40 kDa) was cut out in order to save the antibodies against TASK‐1 channels. This part of the membrane was blocked with 5% nonfat milk (AppliChem, Germany) in TBS (20 mmol·L^−1^ of Tris–HCl, pH 7.6; 150 mmol·L^−1^ of NaCl) with 0.1% Tween 20 (TBSt). Then it was incubated overnight with rabbit polyclonal IgG antibodies against TASK‐1 channels (Antigny et al., 2016) (Abcam Cat# ab49433, RRID:AB_881079, 1:800 in TBSt with 5% BSA). The next day, the membrane was incubated with goat anti‐rabbit IgG secondary antibodies (Cell Signaling Technology Cat# 7074, RRID:AB_2099233, 1:10,000 in 5% milk in TBSt) for 1 h and visualized with SuperSignal West Dura Substrate (Thermo Scientific) using ChemiDoc (Bio‐Rad). Western blotting experiments were analysed using the Image Lab software (Bio‐Rad). The TASK‐1 protein was normalized to loading control (determined by Ponceau S staining) in each sample, and then the median ratio of the adult group was taken as 100%. The Immuno‐related procedures used comply with the recommendations made by the *British Journal of Pharmacology* (Alexander et al., 2018).

### 2.7 BP measurement

Haemodynamic measurements were performed in urethane‐anaesthetized rats (1.2 g·kg^−1^, i.p.). Urethane is widely used as anaesthesia in acute experiments with a terminal point, and it provides long‐term (1 h or more) and stable anaesthesia in rats, including 1‐week‐old pups (Mochalov et al., 2018). Other types of anaesthesia do not match these requirements and may exert undesirable side effects. For example, isoflurane was shown to activate some potassium channels, including several K2P family members (Patel et al., 1999). The use of ketamine in laboratory practice is prohibited in the Russian Federation (Governmental Decree N681 from June 30, 1998). Successful anaesthesia throughout the experiment was indicated as the absence of pupil and flexion reflexes with a potentially painful effect on the foot.

For arterial BP measurements, a polyethylene catheter (PE5‐PE50) was inserted into the left carotid artery. For drug injections, two catheters (PE5‐PE50) were inserted in parallel into the left jugular vein. Body temperature was maintained at 36–37°C with a heating blanket throughout the experiment. Arterial BP was continuously sampled at 1,000 Hz using a BLPR2 transducer (World Precision Instruments, USA) and an USB‐6211 analog‐to‐digital converter (National Instruments, USA). Data recording and processing were performed using a custom‐made software written in LabVIEW 2011 (National Instruments, RRID:SCR_014325). To avoid clotting, the arterial catheter was continuously flushed with heparinized saline (50 U·ml^−1^) using a Syringe pump (Model 341, SAGE Instruments, USA); the infusion rate was 0.2 ml·h^−1^ (for adult rats) or 0.08 ml·h^−1^ (for young rats).

During the initial 10–15 min, arterial pressure and heart rate (HR) were allowed to stabilize. Then the ganglionic blocker chlorisondamine (2.5 mg·kg^−1^, i.v.) was injected through a venous catheter, and a 10‐ to 15‐min‐long baseline recording was obtained. Then AVE1231 (4 mg·kg^−1^, i.v.) or its solvent DMSO (0.5 ml·kg^−1^) was injected through the second venous catheter, and the recording was continued for more than 15 min.

The BP signal was processed beat‐to‐beat to estimate mean arterial pressure (MAP) and HR. Then MAP and HR were averaged in 1.5‐ to 2‐min‐long intervals: (i) before chlorisondamine administration; (ii) before AVE1231 or DMSO administration; and (iii) at the peak response to AVE1231 or the corresponding time period after administration of DMSO.

### 2.8 Drugs

Noradrenaline, ACh, methoxamine (all dissolved in H_2_O), and all salts (American Chemical Society [ACS] grade, for analysis) were obtained from Sigma. DPO‐1 (dissolved in DMSO) and chlorisondamine (dissolved in 0.9% NaCl) were obtained from Tocris. AVE1231 (dissolved in DMSO) was a kind gift of Sanofi.

### 2.9 Data and statistical analysis

The data and statistical analysis comply with the recommendations of the *British Journal of Pharmacology* on experimental design and analysis in pharmacology (Curtis et al., 2018). Treatment of arterial segments with certain substances within each experimental group was randomized. Blinding of the operator was not feasible because vessel responses observed by the operator to manage the experiment permitted inferences about the treatment. However, data analysis was performed semi‐blinded by an independent analyst.

The number of experiments was selected during experimental design based on considerations not to employ an unnecessary amount of animals (3R principles) and to get evidence for an effect of reasonable size in both age groups. Based on the experience of previous studies, the number of animals/tissue samples in each group was at least 6, except for one sample in Figure S1 with *n* = 5. Our previous experiments showed that haemodynamic and vasomotor responses in young rats can be more variable; therefore, we planned and performed more experiments in the young than in adult group, and therefore, adult and young groups are not of equal size. The group size is the number of independent values, and statistical analysis was done using these independent values. Outliers were included in data analysis and presentation.

Statistical analysis was performed using GraphPad Prism 7.0 (La Jolla, CA, USA, RRID:SCR_002798). The normality of the data distribution was tested using the Shapiro–Wilk test. Data are presented as mean and SD (if data distribution was normal) or as median and the interquartile range (if data distribution was different from normal); *n* represents the number of animals (wire myograph, membrane potential, and BP data) or tissue samples (mRNA and protein contents).

Concentration–response relationships to methoxamine between two groups were compared using repeated measures ANOVA. Statistical analyses of values of vessel diameter, force, membrane potential, mRNA contents, and protein contents were performed using a two‐sided unpaired Student's *t* test or Mann–Whitney *U* test, depending on the type of data distribution. Differences were accepted as statistically significant if the *P* value was less than 0.05.

### 2.10 Nomenclature of targets and ligands

Key protein targets and ligands in this article are hyperlinked to corresponding entries in http://www.guidetopharmacology.org and are permanently archived in the Concise Guide to PHARMACOLOGY 2019/20 (Alexander, Christopoulos, et al., 2019; Alexander, Mathie, et al., 2019).

## 3 RESULTS

### 3.1 Expression pattern of K2P channels in saphenous arteries of adult and young rats

**FIGURE 1:**
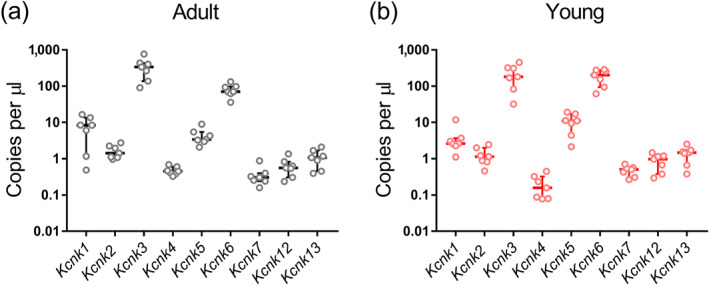
mRNA expression of K2P members in endothelium‐intact saphenous arteries from adult (a, *n* = 7) and young (b, *n* = 7) rats. *Kcnk1*, TWIK‐1; *Kcnk2*, TREK‐1; *Kcnk3*, TASK‐1; *Kcnk4*, TRAAK; *Kcnk5*, TASK‐2; *Kcnk6*, TWIK‐2; *Kcnk7*, TWIK‐3; *Kcnk12*, THIK‐2; *Kcnk13*, THIK‐1. mRNA of *Kcnk9* (TASK‐3), *Kcnk10* (TREK‐2), *Kcnk15* (TASK‐3), and *Kcnk18* (TRESK‐2) was not detected. Data are presented as the median and interquartile range

At the first step of the study, we evaluated the abundance of different K2P family members in arterial tissue on the mRNA level using dPCR. In endothelium‐intact saphenous arteries of adult rats (*n* = 7), mRNA for *Kcnk1*–*Kcnk7*, *Kcnk12*, and *Kcnk13* was expressed (Figure 1a). Among them, mRNA for *Kcnk3* and *Kcnk6* was the most abundant (Figure 1a). Next, we measured the mRNA expression of K2P family members in endothelium‐intact saphenous arteries (*n* = 7) of young rats. Similarly, mRNA for *Kcnk1*–*Kcnk7*, *Kcnk12*, and *Kcnk13* was expressed (Figure 1b); the highest expression level was found for *Kcnk3* and *Kcnk6* (Figure 1b). mRNA for *Kcnk9*, *Kcnk10*, *Kcnk15*, *Kcnk16*, and *Kcnk18* was not detected in arterial samples from either adult or young animals. The functionality of the qPCR assays for these *Kcnk* genes was demonstrated by positive qPCR results from cDNA of rat brain for all *Kcnk* genes except *Kcnk18*. The use of seven different qPCR assays for different *Kcnk18* regions confirmed the negative result and indicated a very low or absent expression of *Kcnk18* in rat brain. Assuming functionality, the commercial TaqMan *Kcnk18* qPCR assay was used for expression analysis in the arterial samples.

### 3.2 Effects of AVE1231 and DPO‐1 on arterial contractile responses in adult and young rats

The next steps in our study were focused on TASK‐1 (*Kcnk3*) channels since this channel (i) is one of the most abundant in saphenous arteries in both age groups; (ii) is one of the most studied in the circulatory system; and (iii) is one of the few K2P channels for which a tool for functional studies is available (TASK‐1 channel blocker AVE1231/A293) (Antigny et al., 2016; Kiper et al., 2015).

The inner normalized diameter (d_100_) of saphenous arteries was 625 ± 87 μm for adult (*n* = 10) and 271 ± 24 μm for young rats (*n* = 16, *P* < 0.05, unpaired Student's *t* test). Maximum active wall tension was 8.3 ± 2.1 mN·mm^−1^ for adult (*n* = 10) and 2.1 ± 0.7 mN·mm^−1^ for young animals (*n* = 16, *P* < 0.05, unpaired Student's *t* test). Importantly, the first concentration–response relationships to methoxamine for endothelium‐denuded arterial segments further treated with potassium channel blockers or solvent, respectively, were not different within the same age group (Table 1).

**TABLE 1:** pD_2_ and maximum active force values of the first concentration–response relationships to methoxamine (preceding the application of either blocker or solvent) in different series of experiments in arteries from adult and young rats

Series of experiments	pD_2_		Maximum active force (mN)	
	Adult	Young	Adult	Young
AVE1231 (*n* = 6, 9)	5.97 ± 0.14	5.69 ± 0.26	36 ± 7	8.3 ± 1.1
DPO‐1 (*n* = 6, 8)	6.00 ± 0.15	5.71 ± 0.27	37 ± 7	8.4 ± 2.3
DPO‐1 + AVE1231 (*n* = 6, 8)	5.99 ± 0.17	5.70 ± 0.23	35 ± 8	8.9 ± 1.7
Time‐control (*n* = 6, 9)	5.94 ± 0.26	5.71 ± 0.20	29 ± 10	8.6 ± 2.1

*Note*: Data are presented as mean ± SD.

**FIGURE 2:**
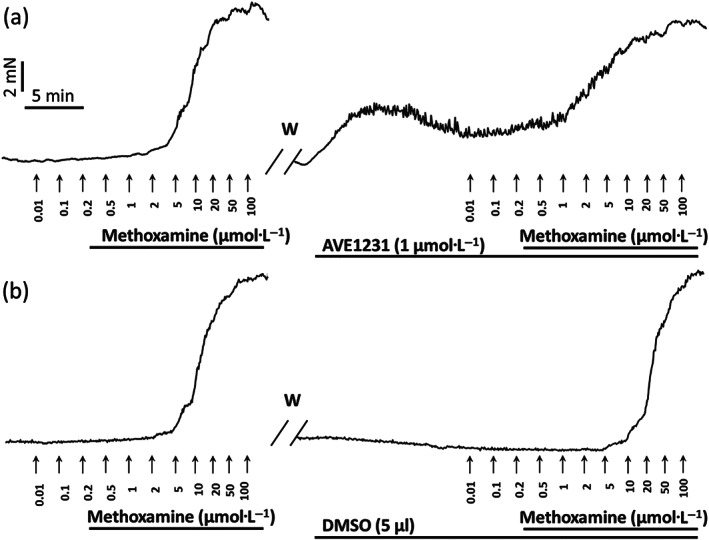
Recordings of contractile responses of two saphenous artery segments to methoxamine in a 12‐day‐old rat. The first concentration–response relationships (left panels in a and b) are similar for the two arterial segments. Application of the TASK‐1 channel blocker AVE1231 (1 μmol·L^−1^) causes an increase in basal tone and of the contractile responses to methoxamine (a), while incubation with an equivalent volume of the solvent (5‐μl DMSO per 5‐ml myograph chamber) does not affect the level of basal tone and the contractile response (b). W, washout

**FIGURE 3:**
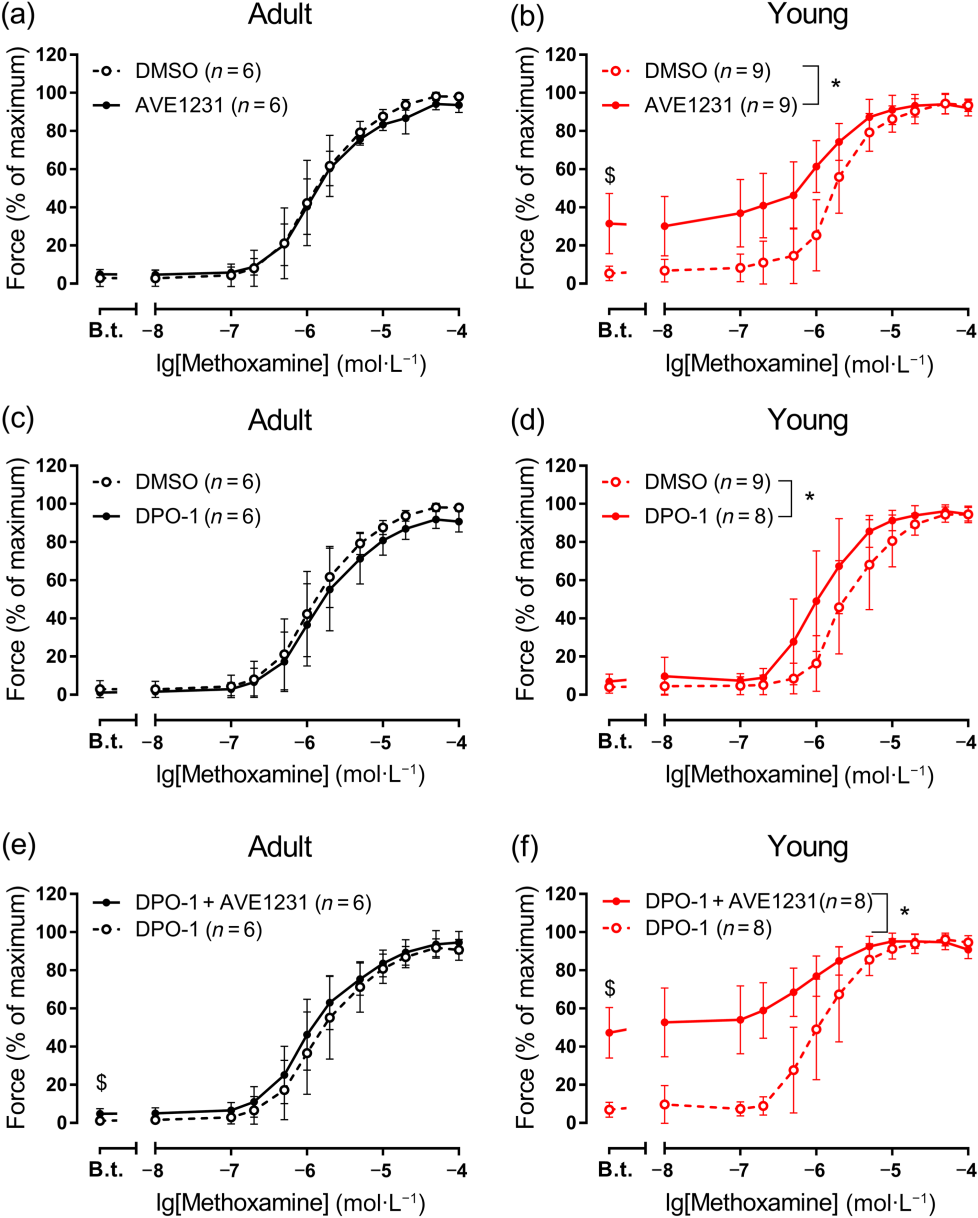
Effects of AVE1231, DPO‐1, and their combined effects on the contractile responses to methoxamine of arteries from two age groups. (a, b) Concentration–response relationships to methoxamine in the presence of solvent (DMSO) or the TASK‐1 channel blocker (AVE1231, 1 μmol·L^−1^) of arteries from adult (a; *n* = 6, 6) and young rats (b; *n* = 9, 9), respectively. (c, d) Concentration–response relationships to methoxamine in the presence of solvent (DMSO) or the Kv1 channel blocker (DPO‐1, 1 μmol·L^−1^) of arteries from adult (c; *n* = 6, 6) and young (d; *n* = 9, 8) rats, respectively. (e, f) Concentration–response relationships to methoxamine in the presence of Kv1 channel blocker only (DPO‐1, 1 μmol·L^−1^) or the Kv1 channel blocker together with TASK‐1 channel blocker (DPO‐1 + AVE1231, both 1 μmol·L^−1^) of arteries from adult (e; *n* = 6, 6) and young (f; *n* = 8, 8) rats, respectively. B.t., basal tone value (active force before the first concentration of methoxamine). Data are presented as mean ± SD. *P < 0.05 between AVE1231 and DMSO or DPO‐1 + AVE1231 and DPO‐1 application (repeated measures ANOVA). ^$^ *P* < 0.05 between basal tone values of AVE1231 and DMSO or DPO‐1 + AVE1231 and DPO‐1 application (unpaired Student's *t* test)

Blockade of TASK‐1 channels with 1 μmol·L^−1^ of AVE1231 (Kiper et al., 2015) did not change the level of basal tone and contractile responses to methoxamine in arteries of adult rats (Figure 3a). However, in arteries of young rats, 1 μmol·L^−1^ of AVE1231 caused the development of a basal tone (about 30% of maximum force) (Figures 2 and 3b). This effect was quite variable and sometimes was accompanied by a small decline with time (Figure 2). The level of active force developed by arteries of young rats during application of methoxamine was also increased by TASK‐1 channel blockade with AVE1231 (Figures 2 and 3b). These data indicate that TASK‐1 channels are important for the regulation of arterial tone in young, but not in adult animals.

Of note, AVE1231 was originally described as a Kv1.5 channel blocker (Ehrlich, Ocholla, Ziemek, & Ru, 2008; Wirth et al., 2007). Thus, its effect on vessels from young rats might be possibly related to its effect on Kv1.5 channels. In order to test this possibility, we performed additional control experiments using the Kv1.5 channel blocker DPO‐1 (Fancher et al., 2015; Lagrutta et al., 2006). DPO‐1 (1 μmol·L^−1^) did not alter basal tone and contractile responses to methoxamine of arteries from adult animals (Figure 3c). Further, AVE1231 in the presence of DPO‐1 slightly elevated basal tone and did not change the contractile responses to methoxamine in arteries of adult animals (Figure 3e). In arteries from young rats, DPO‐1 did not affect basal tone but increased methoxamine‐induced contractions (Figure 3d). Importantly, AVE1231 in the presence of DPO‐1 strongly augmented basal tone and contractions to methoxamine of arteries from young animals (Figure 3f). These data show that the effects of AVE1231 in arteries of young rats remain after blockade of Kv1.5 channels and, therefore, are considered to be predominantly associated with its influence on TASK‐1 channels.

### 3.3 Effects of AVE1231 on smooth muscle cell membrane potential in adult and young rats

**FIGURE 4:**
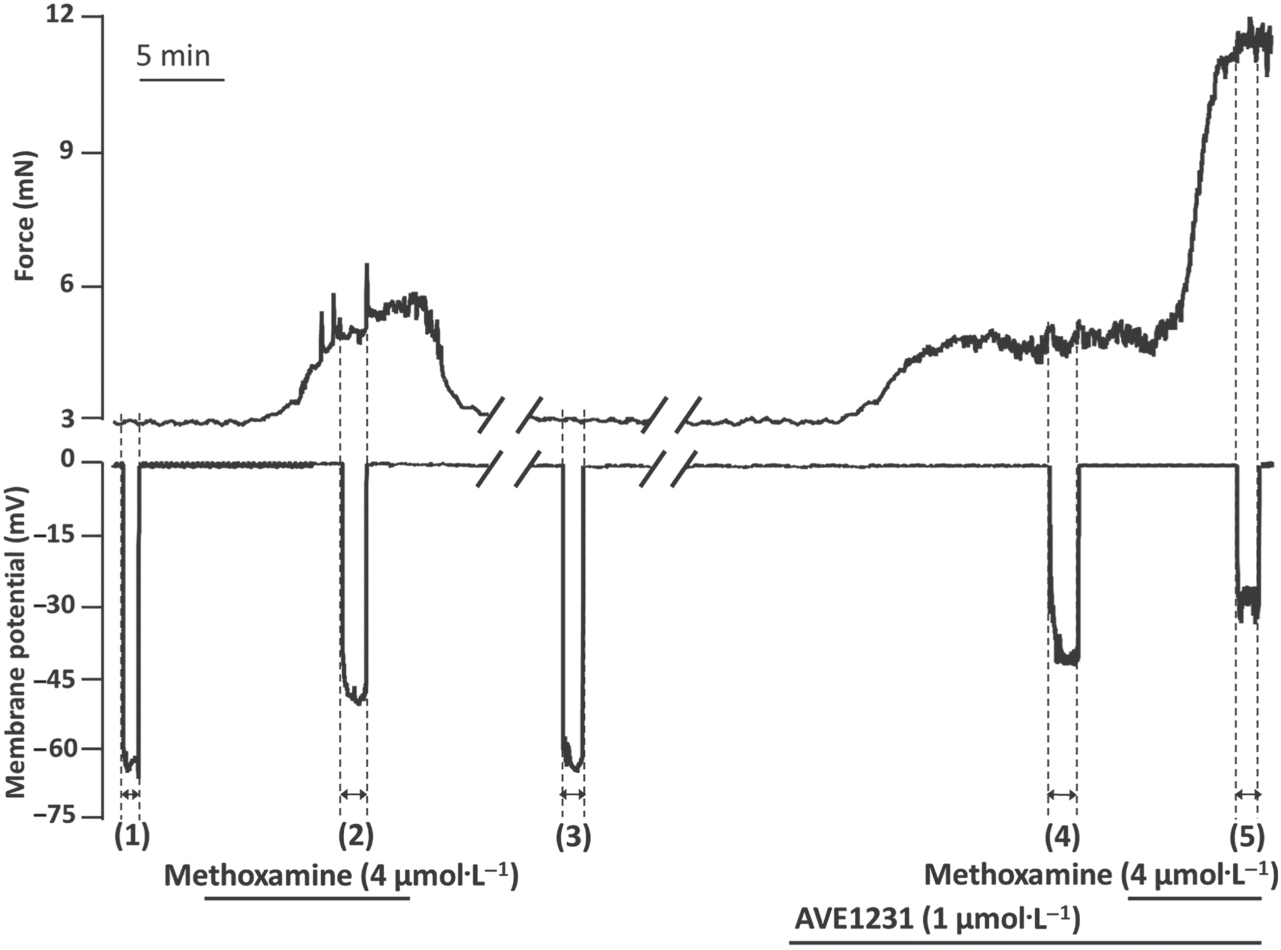
Traces of an experiment with simultaneous registration of force and membrane potential of a saphenous artery from a 12‐day‐old‐rat. Membrane potential and force were detected under five experimental conditions: (1) in the absence of any blockers or agonists—Baseline 1; (2) during methoxamine‐induced contraction (about 20% of maximum force); (3) after washout of methoxamine—Baseline 2; (4) in the presence of AVE1231 (1 μmol·L^−1^); and (5) in the presence of AVE1231 (1 μmol·L^−1^) together with the same concentration of methoxamine as at Step 2

The resting membrane potential (Step 1 in Figure 4, combined data for AVE1231 and time‐control experiments) was −65.0 ± 5.7 mV (*n* = 14) and −67.0 ± 3.4 mV (*n* = 16) in endothelium‐denuded saphenous arteries of adult and young animals respectively (*P* = 0.26, unpaired Student's *t* test).

**FIGURE 5:**
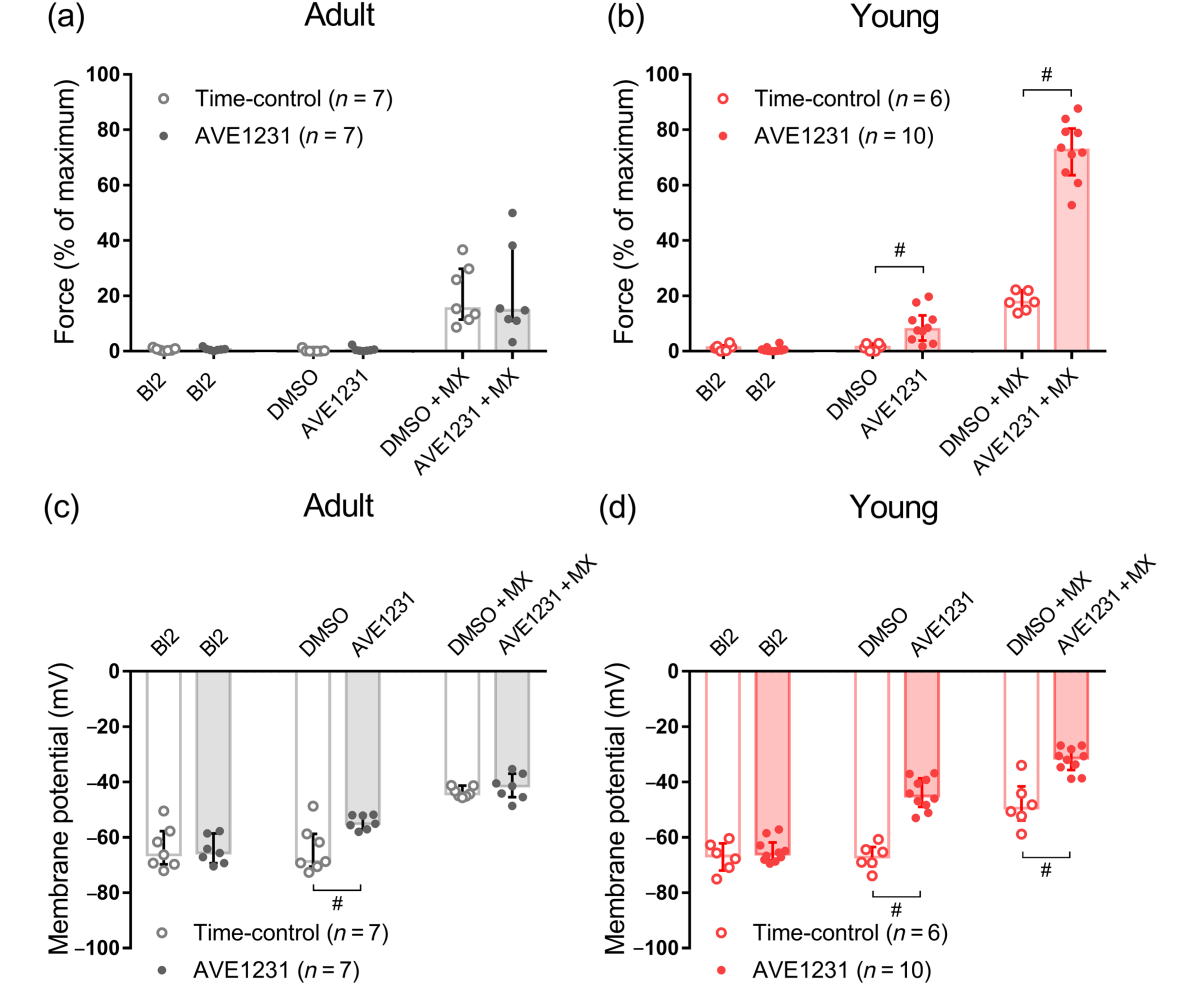
The effects of AVE1231 on force and membrane potential in arterial smooth muscle of two age groups. (a, b) The values of force at baseline (Bl2, corresponds to Baseline 2 in Figure 4), solvent (DMSO) or TASK‐1 channel blocker (AVE1231, 1 μmol·L^−1^), and under the combination of methoxamine (MX) and solvent (DMSO + MX) or AVE1231 (AVE1231 + MX) in saphenous arteries of adult (a; *n* = 7, 7) and young rats (b; *n* = 6, 10), respectively. (c, d) The corresponding values of membrane potential in arteries of adult (c) and young (d) rats, respectively. Data are presented as the median and interquartile range. ^#^ *P* < 0.05 between time‐control and AVE1231 application (Mann–Whitney *U* test)

Importantly, the values of force and membrane potential at Step 1 as well as the methoxamine‐induced depolarization and increase in active force (Step 2 in Figure 4; see Section 2 for detailed description) did not differ between preparations further treated with AVE1231 or solvent, respectively, within the same age group (Table 2). This setting allowed us to estimate the effects of AVE1231 by comparing the values at Steps 3–5 between AVE1231‐ and DMSO‐treated vessels (these data are shown in Figure 5a–d).

**TABLE 2:** Changes of force and membrane potential induced by methoxamine during the first application (Step 2 in Figure 4) in experiments with either blocker (AVE1231) or vehicle (DMSO) application in arteries from adult and young rats

Parameter	Adult		Young	
	Before AVE1231 (*n* = 7)	Time‐control (*n* = 7)	Before AVE1231 (*n* = 10)	Time‐control (*n* = 6)
Change of force (% of maximum)	19.1 ± 14.6	14.8 ± 8.4	17.3 ± 9.9	18.2 ± 3.6
Change of membrane potential (mV)	24.0 ± 4.7	20.5 ± 6.3	20.0 ± 10.7	19.6 ± 12.7

*Note*: Data are presented as mean ± SD.

AVE1231 did not change basal tone in arteries of adult rats (Figure 5a) but induced smooth muscle depolarization (Figure 5c). In arteries of adult animals, the values of active force and membrane potential during methoxamine application were similar in the presence of AVE1231 or solvent (Figure 5a,c). In contrast, in arteries of young animals, AVE1231 induced strong basal tone (Figures 4 and 5b) associated with pronounced smooth muscle depolarization (Figures 4 and 5d). Importantly, the AVE1231‐induced depolarization (identified as the difference between Step 4 and Step 3 in AVE1231‐treated preparations; see Figure 4 and Section 2 for description) was considerably larger for young rats (19.1 [16.6–26.5] mV, *n* = 10) compared with adult animals (12.3 [5.7–15.4] mV, *n* = 7, *P* < 0.05, Mann–Whitney *U* test). Further, in young rats, AVE1231 considerably increased methoxamine‐induced contraction and led to higher depolarization compared with time‐control preparations (Figures 4 and 5b,d). Taken together, these data demonstrate that the stronger influence of TASK‐1 channel blockade on basal tone and methoxamine‐induced contractile responses in arteries of young rats compared with older animals is associated with its higher impact on membrane potential.

### 3.4 Comparison of TASK‐1 channel abundance in endothelium‐denuded arteries of adult and young rats

**FIGURE 6:**
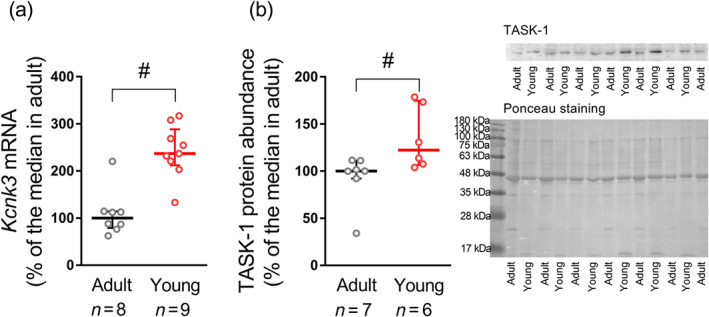
TASK‐1 channel abundance in endothelium‐denuded arteries of two age groups. (a) Relative expression levels of *Kcnk3* pore‐forming subunit mRNA in arteries from adult (*n* = 8) and young (*n* = 9) rats. Data are normalized to the geometric mean of *Rn18s* and *Gapdh*. (b) Protein content of TASK‐1 pore‐forming subunit in arteries from adult (*n* = 7) and young (*n* = 6) rats. Data are normalized to loading control (determined by Ponceau S staining). The median value of TASK‐1 mRNA or protein content in the adult group is taken as 100%. Data are presented as the median and interquartile range. ^#^ *P* < 0.05 between adult and young animals (Mann–Whitney *U* test)

In order to identify whether the observed stronger impact of TASK‐1 channels on smooth muscle contractile responses and depolarization in the younger age group is associated with their higher abundance in arterial smooth muscle, we estimated mRNA and protein contents of TASK‐1 in *endothelium‐denuded* arteries of the two age groups. Indeed, the relative expression level of the *Kcnk3* gene in arterial preparations was considerably higher in young compared with adult animals (Figure 6a). Further, protein abundance of TASK‐1 channels was larger in smooth muscle of young compared with adult animals (Figure 6b). Thus, our results clearly demonstrate that the larger role of TASK‐1 channels at the functional level in arteries of young rats is associated with their higher expression level.

### 3.5 Effects of AVE1231 on systemic cardiovascular parameters in adult and young rats

To assess the role of TASK‐1 channels in the regulation of systemic haemodynamics, we evaluated the effects of AVE1231 on MAP and HR in anaesthetized rats. At the beginning of the experiment, MAP was almost two times lower in young compared with adult animals; HR did not differ between the two age groups (Table S1). In order to exclude the influence of the autonomic nervous system, the effects of AVE1231 were studied after ganglionic blockade by chlorisondamine (2.5 mg·kg^−1^). After ganglionic blockade, both MAP and HR values were lower in young compared with adult rats (Table S1).

**FIGURE 7:**
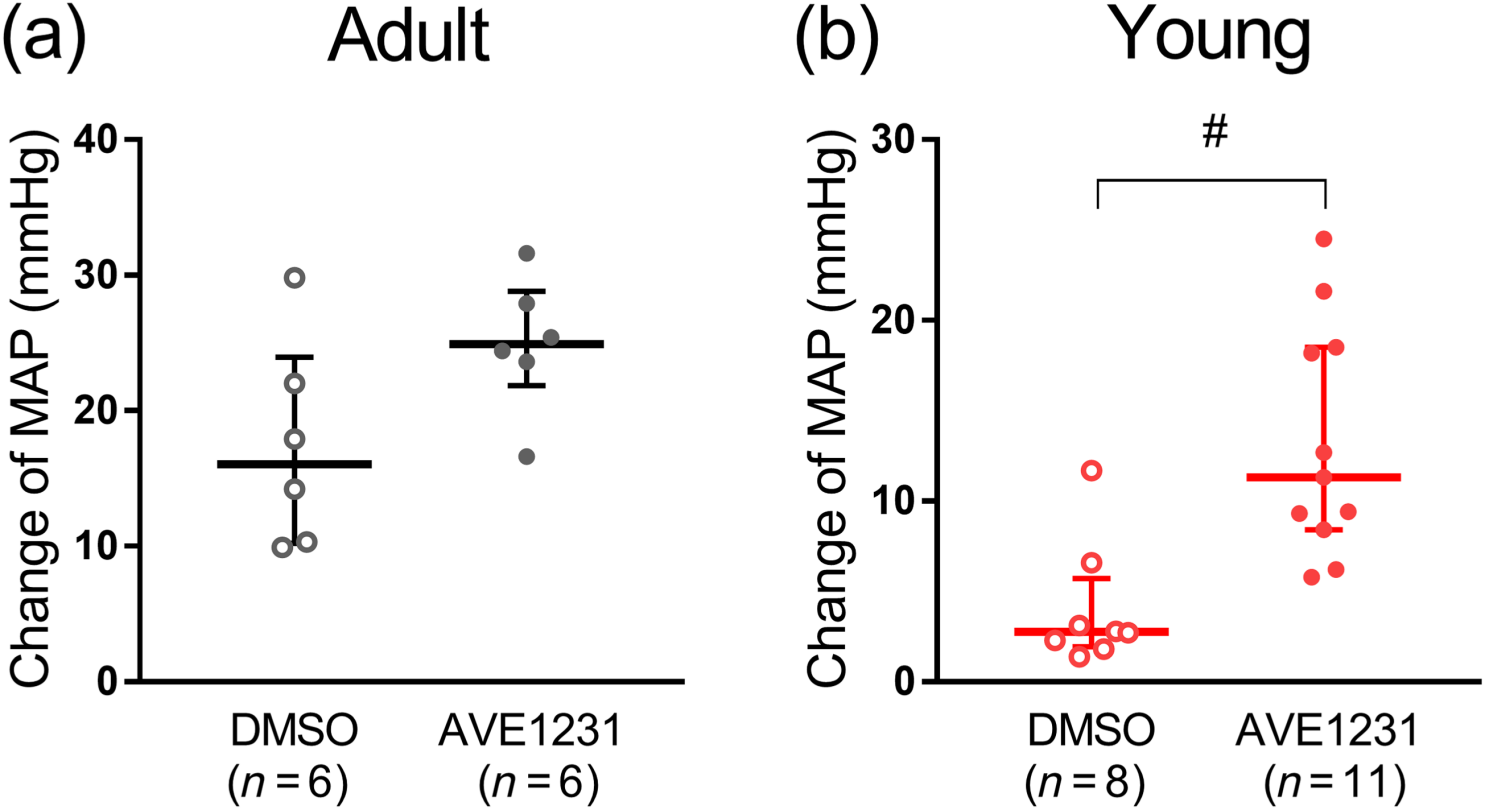
AVE1231 increases mean arterial pressure (MAP) in young (b) but not adult (a) animals. The change of MAP was calculated as the difference between the peak MAP value after intravenous administration of vehicle (DMSO, 0.5 ml·kg^−1^) or AVE1231 (4 mg·kg^−1^) and the respective baseline value. The experiments were performed under the condition of ganglionic blockade (chlorisondamine, 2.5 mg·kg^−1^). Data are presented as the median and interquartile range. ^#^ *P* < 0.05 between DMSO and AVE1231 experiments (Mann–Whitney *U* test)

In each age group, some of the animals were further treated with AVE1231 and others with the vehicle (DMSO). Importantly, no differences in baseline values of either MAP or HR were observed between the two subgroups (further treated with AVE1231 or with DMSO) within the same age group (Table S2). In adult rats, AVE1231 induced a transient increase of MAP that was similar to the changes in the DMSO‐treated subgroup (Figure 7a). In contrast, in young rats, the AVE1231‐induced MAP increase was larger compared with the DMSO‐treated subgroup (Figure 7b). The effect of AVE1231 on MAP plateaued after 2–3 min. Noteworthy, HR levels after administration of AVE1231 were not different compared with their levels in the DMSO‐treated subgroups in both adult and young animals (Table S2). Thus, TASK‐1 blockade increased the systemic BP level only in young rats, and this effect was not associated with an altered HR.

## 4 DISCUSSION

This study provides novel data on developmental alterations in the functioning of TASK‐1 channels in arterial smooth muscle. We showed that smooth muscle mRNA and protein content of TASK‐1 channels is higher in peripheral arteries from young compared with adult rats. In accordance with this, TASK‐1 channels oppose vasocontraction by counteracting arterial smooth muscle depolarization only at early postnatal age but not in adulthood. Moreover, TASK‐1 channel blockade affected systemic BP in young but not adult rats.

### 4.1 TASK‐1 channels are highly abundant in rat saphenous arteries

A number of studies have demonstrated expression of some K2P channel members in cerebral (Bryan et al., 2006) as well as pulmonary and mesenteric arteries (Gardener et al., 2004) of rats. To the best of our knowledge, for the first time, we evaluated the expression pattern of all known K2P channel family members in a rat artery, the saphenous artery, using the dPCR approach. The advantage of dPCR is that it enables to estimate the number of single mRNA molecules in tissue samples regardless of primer efficiency. The latter allows the quantitative comparison of the mRNA contents of different K2P channel family members. The results of our screening demonstrate that saphenous arteries of both adult and young rats contain a large number of TWIK‐2 and TASK‐1 mRNA. Noteworthy, using dPCR, we did not compare the content of K2P channel family members between adult and young rats since the data for two age groups came from two different experimental series.

### 4.2 TASK‐1 channels are more abundant in smooth muscle cells of young compared with adult rats

Since the TASK‐1 channel is one of the most abundant K2P channel family member in the rat saphenous artery, developmental alterations of TASK‐1 expression in rat saphenous arteries were evaluated using standard qPCR and Western blot techniques. We showed for the first time that the contents of TASK‐1 channel mRNA and protein are higher in arteries of young rats compared with adult animals. Similarly, previously, we demonstrated increased expression levels of Kv7.4 channels and its accessory KCNE4 subunit in arteries of 10‐ to 15‐day‐old rats (Shvetsova et al., 2019). These observations point to alterations in the expression pattern of several potassium channels in the developing vasculature. Noteworthy, all these studies were performed on arteries with denuded endothelium, so that the expression of the channel was determined mainly for arterial smooth muscle cells of the two age groups.

### 4.3 TASK‐1 channels are important for the regulation of membrane potential and arterial tone in arteries of young rats

The ability of K2P channels to conduct outward potassium currents in a wide potential range suggests their important role in the regulation of membrane potential and, therefore, vascular tone (Gurney & Manoury, 2009). Indeed, a number of studies have demonstrated a pronounced anticontractile influence of these channels in pulmonary arteries of human and rat (Antigny et al., 2016; Gardener et al., 2004). However, a functional role of TASK‐1 channels in pulmonary arteries of mice was not observed (Manoury, Lamalle, Oliveira, Reid, & Gurney, 2011; Murtaza et al., 2017). We also did not observe considerable effects of AVE1231 on contractile responses in saphenous arteries of adult rats, despite it caused small subthreshold depolarization of their smooth muscle. Similarly, in our previous experiments on the saphenous artery of adult rats, the blockade of Kv7 or Kir channels (by XE991 [3 μmol·L^−1^] or BaCl_2_ [30 μmol·L^−1^], respectively) depolarized arterial smooth muscle by about 7 mV, while it did not induce the development of a basal tone (Shvetsova et al., 2019). In addition, in experiments on rat small mesenteric and tail arteries, an increase of the extracellular potassium concentration up to 20–25 mmol·L^−1^ induced depolarization of smooth muscle cells by 10–15 mV, which was not associated with the development of contractile responses (Mulvany, Nilsson, & Flatman, 1982; Neild & Kotecha, 1987). Therefore, during non‐receptor stimulation, arterial smooth muscle starts to contract only from a certain threshold level of membrane potential. Probably, 12‐mV smooth muscle depolarization induced by AVE1231 in arteries from adult rats was subthreshold for the development of a contractile response.

In contrast, in young rats, blockade of TASK‐1 channels with AVE1231 caused a strong smooth muscle depolarization, which was sufficient for the development of contraction. Previously, we demonstrated a higher contribution of Kir and especially Kv7 channels in the regulation of membrane potential and vascular tone in arteries from young compared with adult rats (Shvetsova et al., 2019). Our new data suggest that TASK‐1 channels together with Kir and Kv7 channels play an important role in the negative feedback regulation of vasocontraction in the period of early postnatal ontogenesis, but not in adulthood.

To our knowledge, in this study, AVE1231 was used to study the role of TASK‐1 channels in rat systemic arteries for the first time. Previously, AVE1231 (A293) at the same concentration (1 μmol·L^−1^) was shown to increase contractile responses of isolated rat pulmonary arteries (Antigny et al., 2016). Of note, the IC_50_ of AVE1231 for human TASK‐1 channels in *Xenopus* oocytes is 0.2 μmol·L^−1^, while its IC_50_ against Kv1.5 is 43 times higher (Kiper et al., 2015). Thus, at the concentration used, AVE1231 would block primarily and considerably TASK‐1 channels but not Kv1.5 channels. This suggestion is supported by our data showing additive effects of AVE1231 (1 μmol·L^−1^) and DPO‐1 (1 μmol·L^−1^) in arteries of young rats. Since the IC_50_ for DPO‐1 against Kv1.5 channels is less than 0.2 μmol·L^−1^ (Lagrutta et al., 2006), 1 μmol·L^−1^ of DPO‐1 should block Kv1.5 channels to a large degree (in a specific manner) (Tsvetkov et al., 2016). Thus, the large additional effect of 1 μmol·L^−1^ of AVE1231 is most likely due to a block of TASK‐1 channels rather than a blockade of the few remaining Kv1.5 channels after DPO‐1 blockade.

Therefore, TASK‐1 channels counteract arterial smooth muscle depolarization and, thereby, reduce contractile responses of arterial smooth muscle in young but not adult rats, in line with the greater expression of TASK‐1 channels in smooth muscle of saphenous arteries at the early postnatal period. Of note, TASK‐1 channels limiting smooth muscle depolarization are most likely located primarily in the smooth muscle cells of the vessel wall since the effects of AVE1231 on basal tone and on contractile responses to methoxamine were comparable in endothelium‐intact and endothelium‐denuded arteries from young rats (Figure S2).

### 4.4 TASK‐1 channels are important for arterial BP control in young rats

To get further insight into the physiological role of TASK‐1 channels, we compared the effects of AVE1231 on MAP in the two age groups of rats. Of note, blood flow to the skin comprises up to 20% of the cardiac output (Štulcová, 1977) and, thereby, is functionally relevant. Therefore, functional alterations of skin feed (saphenous) arteries could affect the BP level in young rats.

In our experiments, AVE1231 was dissolved in DMSO, which is the most often used vehicle for hydrophobic substances. However, DMSO may exert its own biological effects, which could confound the effects of AVE1231 (Kelava, Cavar, & Culo, 2011; Sawada & Sato, 1975). To distinguish between the effects of the blocker and the vehicle, we compared the change of MAP at the same time points in animal groups treated with AVE1231 and DMSO respectively.

In a previous study, Wirth et al. (2007) evaluated the in vivo effects of AVE1231 in pigs and goats using a dose of 3 mg·kg^−1^. In our experiments, we started from a dose of 1 mg·kg^−1^ and then increased it in a stepwise manner to get an evident effect on BP at least in one of the experimental groups. The minimum dose of AVE1231, which affected BP, was 4 mg·kg^−1^. This is close to the dose in the study by Wirth et al. (2007), especially taking into account the differences in body surface area and metabolic rate between pigs/goats and rats (Nair & Jacob, 2016; Reagan‐Shaw, Nihal, & Ahmad, 2008).

The MAP level was almost two times lower in young rats compared with adult animals, which is consistent with previously published data (Mochalov et al., 2018). The intravenous administration of AVE1231 (4 mg·kg^−1^) increased MAP in young rats, but not adult animals. Similarly, loss‐of‐function mutations in TASK‐1 channels did not change systemic arterial pressure in adult rats (Lambert et al., 2019). Therefore, TASK‐1 channels have a depressor effect at the systemic level only in the period of early postnatal development.

It is important to note that our experiments were carried out under conditions of ganglionic blockade. The use of ganglionic blockade allowed us to exclude the influence of AVE1231 on the activity of the autonomic nervous system and, therefore, to evaluate its effects exclusively in the periphery. TASK‐1 channels are abundant in rat brain, including the ventrolateral medulla neurons, which are involved in the autonomic control of the circulation (Kindler, Pietruck, Yost, Sampson, & Gray, 2000; Washburn, Bayliss, & Guyenet, 2003). Of note, the expression of TASK‐1 channels in the rat ventrolateral medulla is developmentally regulated (Kanjhan, Anselme, Noakes, & Bellingham, 2004). Therefore, in the absence of ganglionic blockade, AVE1231 would affect the nervous control of the circulation, which is different in adult and young rats because of the immaturity of the vasomotor innervation in the younger group (Puzdrova et al., 2014).

We did not find differences in HR between AVE1231‐ and DMSO‐treated groups of rats at either age. Previously, no chronotropic effect of AVE1231 in a similar dose (3 mg·kg^−1^) was observed in anaesthetized pigs as well (Wirth et al., 2007). Thus, we suggest that in our experimental conditions, AVE1231 had no prominent effect on heart function.

Taken together, our in vivo observations support the in vitro data demonstrating a more prominent influence of TASK‐1 channels in arteries from young compared with older rats. Based on our results, we suggest that TASK‐1 channels are important regulators of vascular tone and arterial pressure in the early postnatal period. Our BP measurements point to the presence of a TASK‐1 channel‐dependent control of vascular resistance not only in cutaneous but also in other vascular regions.

## 5 CONCLUSION

In conclusion, our novel findings demonstrate a pronounced negative feedback regulation of vasocontraction by TASK‐1 channels in the early postnatal period in rats. Taking into account the growing incidence of primary hypertension in childhood (Flynn, 2018), it is extremely important to identify all particular players regulating vascular tone and BP in the immature organism. We suppose that a large contribution of TASK‐1 channels together with Kir and Kv7 channels (Shvetsova et al., 2019) is important to reduce peripheral vascular resistance and the BP level in the newborn organism to protect the immature arteries from high transmural pressure and the immature heart from high afterload. In addition, a pronounced anticontractile role of TASK‐1 channels may contribute to an increase in the blood supply to growing organs during early postnatal development. It is known that the mechanisms regulating arterial smooth muscle contraction shift from calcium independent to calcium dependent during postnatal maturation (Akopov, Zhang, & Pearce, 1998a, 1998b; Mochalov et al., 2018; Puzdrova et al., 2014). Similarly, the role of potassium channels regulated by calcium to a lesser extent (TASK‐1, Kv7, and Kir) decreases, while the role of calcium‐dependent BKCa channels increases during postnatal development (Shvetsova et al., 2019), which could be a hallmark of maturation of peripheral arteries. The mechanism providing the postnatal decline in the contribution of TASK‐1 channels to the regulation of vascular tone remains elusive and could serve as a topic for future studies.

## AUTHOR CONTRIBUTIONS

A.A.S., D.K.G., N.S., and P.B. performed the experiments. A.A.S., D.K.G., O.S.T., and R.S. analysed the data. E.V.L. developed a program for BP registration. A.A.S., D.K.G., O.S.T., and R.S. wrote the manuscript. A.A.S., D.K.G., N.S., P.B., E.V.L., O.S.T., and R.S. have read and approved the manuscript.

## CONFLICT OF INTEREST

The authors declare no conflicts of interest.

## DECLARATION OF TRANSPARENCY AND SCIENTIFIC RIGOUR

This Declaration acknowledges that this paper adheres to the principles for transparent reporting and scientific rigour of preclinical research as stated in the *BJP* guidelines for Design & Analysis, Immunoblotting and Immunochemistry, and Animal Experimentation and as recommended by funding agencies, publishers, and other organizations engaged with supporting research.

## Supporting information


**Figure S1.** Endothelium removal results in a considerable drop of Nos3 mRNA content in saphenous artery samples from both adult (a) and young (b) animals. Data are normalized to the geometric mean of Rn18s and Tagln, the median value of Nos3 mRNA content in the endothelium‐intact group is taken as 100%. Data are presented as the median and interquartile range. #*P* < 0.05 between endothelium‐intact (Endo+) and endothelium‐denuded (Endo‐) arterial samples (Mann–Whitney U test).

**Figure S2.** Concentration‐response relationships to methoxamine in the presence of solvent (DMSO, *n* = 10) or the TASK‐1 channel blocker (AVE1231, 1 μmol L^−1^, *n* = 6) of arteries with intact endothelium from young rats. Data are presented as mean ± SD. *P < 0.05 between AVE1231 and DMSO (Repeated measures ANOVA). ^$^ *P* < 0.05 between basal tone values of AVE1231 and DMSO (unpaired Student's *t* test).

**Table S1.** Values of mean arterial pressure (MAP) and heart rate (HR) in two age groups of rats before and after administration of chlorisondamine (2.5 mg kg^−1^). Data are presented as the median and interquartile range. ^#^ *P* < 0.05 between Adult and Young groups (Mann–Whitney U test).
Table S2. Baseline and peak treatment values of mean arterial pressure (MAP) and heart rate (HR) in two age groups of rats in experiments with administration of the vehicle (DMSO, 0.5 mL kg^−1^) or AVE1231 (4 mg kg^−1^) under the condition of ganglionic blockade (chlorisondamine, 2.5 mg kg^−1^). Data are presented as the median and interquartile range. ^#^ *P* < 0.05 between DMSO and AVE1231 experiments (Mann–Whitney U test).
