# Supplementary figures and images for: TASK‐1 channel blockade by AVE1231 increases vasocontractile responses and BP in 1‐ to 2‐week‐old but not adult rats
Source: Br J Pharmacol. 2020 Sep 24;177(22):5148–62. doi: 10.1111/bph.15249 (PMC7589011; doi:10.1111/bph.15249)

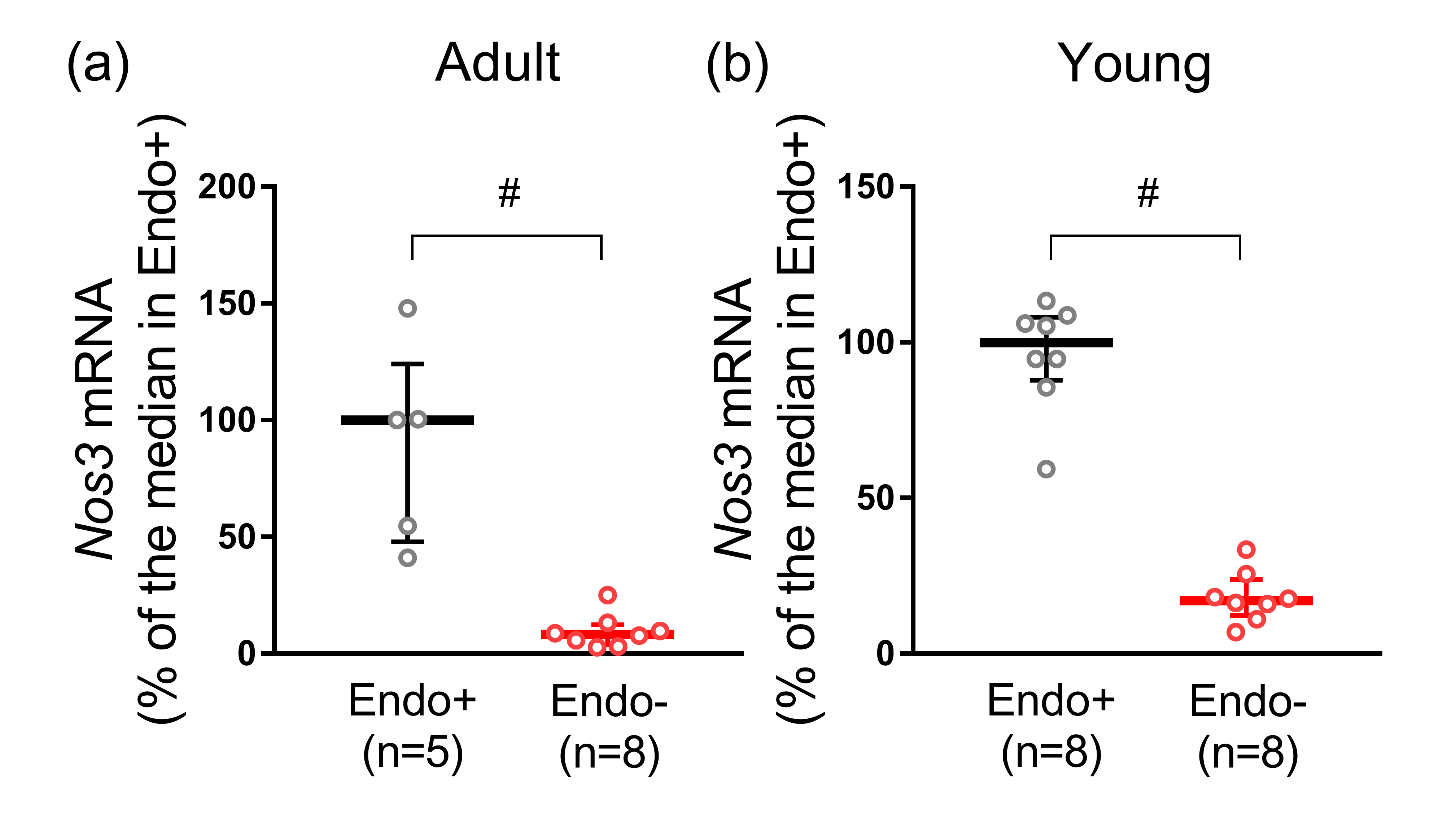

Supplement: Supplementary file 1 — Figure S1. Endothelium removal results in a considerable drop of Nos3 mRNA content in saphenous artery samples from both adult (a) and young (b) animals. Data are normalized to the geometric mean of Rn18s and Tagln, the median value of Nos3 mRNA content in the endothelium‐intact group is taken as 100%. Data are presented as the median and interquartile range. #P < 0.05 between endothelium‐intact (Endo+) and endothelium‐denuded (Endo‐) arterial samples (Mann–Whitney U test). [file BPH-177-5148-s001.tif]

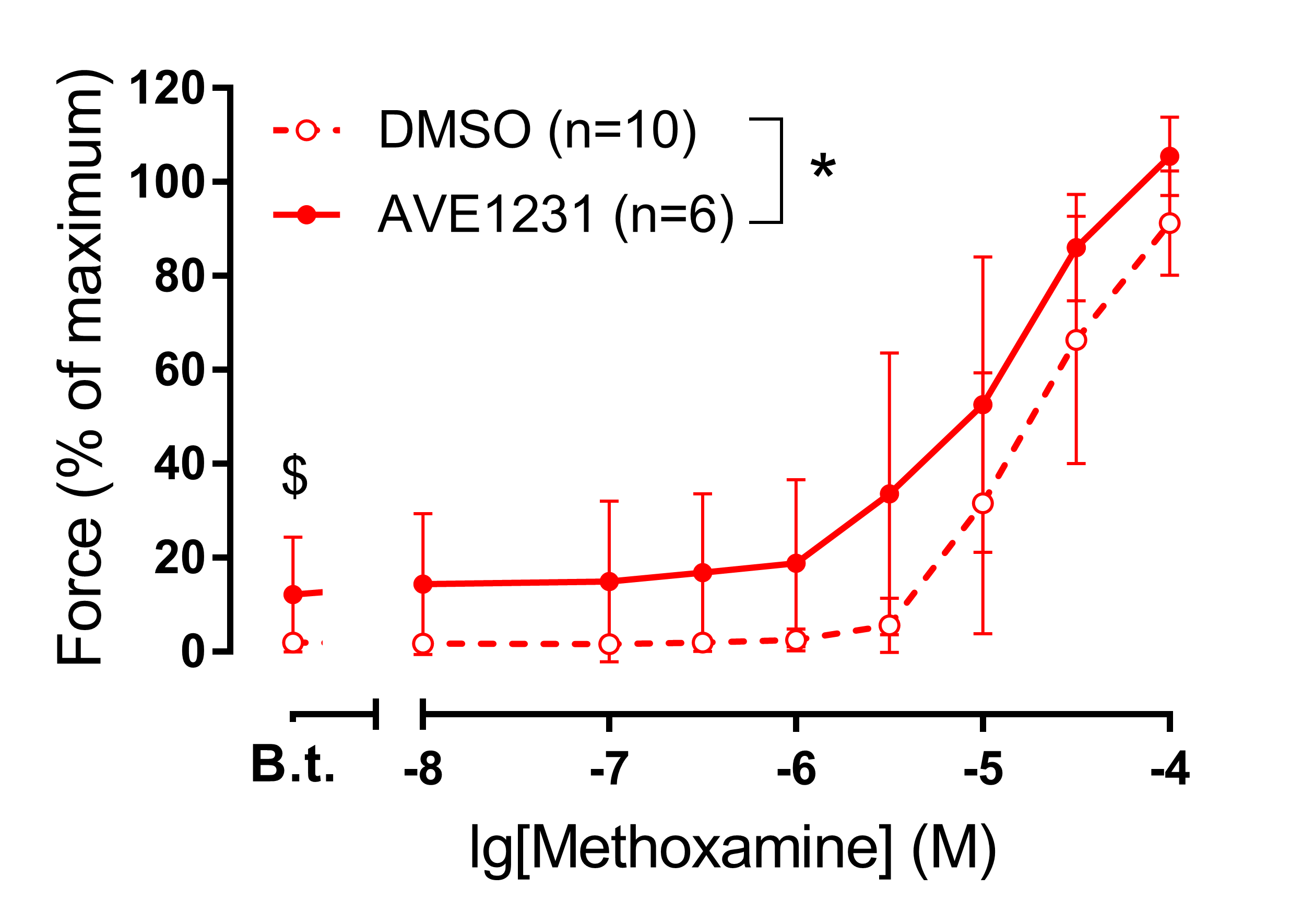

Supplement: Supplementary file 2 — Figure S2. Concentration‐response relationships to methoxamine in the presence of solvent (DMSO, n = 10) or the TASK‐1 channel blocker (AVE1231, 1 μmol L−1, n = 6) of arteries with intact endothelium from young rats. Data are presented as mean ± SD. *P < 0.05 between AVE1231 and DMSO (Repeated measures ANOVA). $ P < 0.05 between basal tone values of AVE1231 and DMSO (unpaired Student's t test). [file BPH-177-5148-s002.tif]
