# Supplementary material for: TASK‐1 channel blockade by AVE1231 increases vasocontractile responses and BP in 1‐ to 2‐week‐old but not adult rats
Source: Br J Pharmacol. 2020 Sep 24;177(22):5148–62. doi: 10.1111/bph.15249 (PMC7589011; doi:10.1111/bph.15249)
Supplement: Supplementary file 3 — Table S1. Values of mean arterial pressure (MAP) and heart rate (HR) in two age groups of rats before and after administration of chlorisondamine (2.5 mg kg−1). Data are presented as the median and interquartile range. # P < 0.05 between Adult and Young groups (Mann–Whitney U test). Table S2. Baseline and peak treatment values of mean arterial pressure (MAP) and heart rate (HR) in two age groups of rats in experiments with administration of the vehicle (DMSO, 0.5 mL kg−1) or AVE1231 (4 mg kg−1) under the condition of ganglionic blockade (chlorisondamine, 2.5 mg kg−1). Data are presented as the median and interquartile range. # P < 0.05 between DMSO and AVE1231 experiments (Mann–Whitney U test). [file BPH-177-5148-s003.docx]

Supplementary Table S1. Values of mean arterial pressure (MAP) and heart rate (HR) in two age groups of rats before and after administration of chlorisondamine (2.5 mg kg^-1^). Data are presented as the median and interquartile range. ^#^P < 0.05 between Adult and Young groups (Mann-Whitney U test).

|  | Adult (n=12) | Young (n=19) |
| --- | --- | --- |
| *Mean Arterial Pressure (mm Hg)* | | |
| Before chlorisondamine | 85.0 (76.8-90.8) | 43.3 (40.4-52.2)^#^ |
| After chlorisondamine | 62.7 (58.8-65.5) | 31.0 (29.9-36.0)^#^ |
| *Heart Rate (bpm)* | | |
| Before chlorisondamine | 340 (317-366) | 333 (298-357) |
| After chlorisondamine | 332 (311-362) | 215 (200-245)^#^ |

Supplement Table S2. Baseline and peak treatment values of mean arterial pressure (MAP) and heart rate (HR) in two age groups of rats in experiments with administration of the vehicle (DMSO, 0.5 mL kg^-1^) or AVE1231 (4 mg kg^-1^) under the condition of ganglionic blockade (chlorisondamine, 2.5 mg kg^-1^). Data are presented as the median and interquartile range. ^#^P < 0.05 between DMSO and AVE1231 experiments (Mann-Whitney U test).

|  | Adult | | Young | |
| --- | --- | --- | --- | --- |
|  | DMSO (n=6) | AVE1231  (n=6) | DMSO (n=8) | AVE1231  (n=11) |
| *Mean Arterial Pressure (mm Hg)* | | | | |
| Baseline | 63.6 (57.0-68.0) | 61.8 (59.4-63.8) | 30.4 (29.0-33.5) | 31.9 (29.9-36.9) |
| DMSO/AVE1231 | 81.4 (74.2-85.0) | 85.9 (83.7-91.0) | 33.8 (33.0-35.2) | 42.0 (38.1-58.5)^#^ |
| *Heart Rate (bpm)* | | | | |
| Baseline | 329 (308-367) | 332 (311-378) | 212 (199-240) | 228 (200-282) |
| DMSO/AVE1231 | 337 (321-381) | 347 (326-423) | 219 (201-242) | 237 (231-300) |
